# Epigallocatechin-3-Gallate Accelerates Relaxation and Ca^2+^ Transient Decay and Desensitizes Myofilaments in Healthy and *Mybpc3*-Targeted Knock-in Cardiomyopathic Mice

**DOI:** 10.3389/fphys.2016.00607

**Published:** 2016-12-05

**Authors:** Felix W. Friedrich, Frederik Flenner, Mahtab Nasib, Thomas Eschenhagen, Lucie Carrier

**Affiliations:** ^1^Cardiovascular Research Center, Department of Experimental Pharmacology and Toxicology, University Medical Center Hamburg-EppendorfHamburg, Germany; ^2^German Centre for Cardiovascular Research (DZHK)Hamburg, Germany

**Keywords:** epigallocatechin-3-gallate, hypertrophic cardiomyopathy, *Mybpc3*, myofilament Ca^2+^ sensitivity, relaxation, Ca^2+^ transient

## Abstract

**Background:** Hypertrophic cardiomyopathy (HCM) is the most common inherited cardiac muscle disease with left ventricular hypertrophy, interstitial fibrosis and diastolic dysfunction. Increased myofilament Ca^2+^ sensitivity could be the underlying cause of diastolic dysfunction. Epigallocatechin-3-gallate (EGCg), a catechin found in green tea, has been reported to decrease myofilament Ca^2+^ sensitivity in HCM models with troponin mutations. However, whether this is also the case for HCM-associated thick filament mutations is not known. Therefore, we evaluated whether EGCg affects the behavior of cardiomyocytes and myofilaments of an HCM mouse model carrying a gene mutation in cardiac myosin-binding protein C and exhibiting both increased myofilament Ca^2+^ sensitivity and diastolic dysfunction.

**Methods and Results:** Acute effects of EGCg were tested on fractional sarcomere shortening and Ca^2+^ transients in intact ventricular myocytes and on force-Ca^2+^ relationship of skinned ventricular muscle strips isolated from *Mybpc3*-targeted knock-in (KI) and wild-type (WT) mice. Fractional sarcomere shortening and Ca^2+^ transients were analyzed at 37°C under 1-Hz pacing in the absence or presence of EGCg (1.8 μM). At baseline and in the absence of Fura-2, KI cardiomyocytes displayed lower diastolic sarcomere length, higher fractional sarcomere shortening, longer time to peak shortening and time to 50% relengthening than WT cardiomyocytes. In WT and KI neither diastolic sarcomere length nor fractional sarcomere shortening were influenced by EGCg treatment, but relaxation time was reduced, to a greater extent in KI cells. EGCg shortened time to peak Ca^2+^ and Ca^2+^ transient decay in Fura-2-loaded WT and KI cardiomyocytes. EGCg did not influence phosphorylation of phospholamban. In skinned cardiac muscle strips, EGCg (30 μM) decreased Ca^2+^ sensitivity in both groups.

**Conclusion:** EGCg hastened relaxation and Ca^2+^ transient decay to a larger extent in KI than in WT cardiomyocytes. This effect could be partially explained by myofilament Ca^2+^ desensitization.

## Introduction

Hypertrophic cardiomyopathy (HCM) is the most common cardiac genetic disease, with more than 1400 different mutations in genes encoding primarily sarcomeric proteins (Friedrich and Carrier, 2012; Maron et al., 2014; Ho et al., 2015). The most frequently mutated genes are *MYH7* (encoding β-myosin-heavy chain) and *MYBPC3* (encoding cardiac myosin-binding protein C), which constitute about 80% of known mutations. Besides a typical hypertrophy of the left ventricle, patients often present a normal or increased ejection fraction, but a compromised diastolic function with an incomplete relaxation and increased filling pressures (Elliott et al., 2014). Diastolic dysfunction may result in left atrial enlargement and is associated with exercise intolerance and bad prognosis in HCM, primarily due to supraventricular arrhythmias (Yang et al., 2009). Tissue Doppler measurements have revealed that a reduction in systolic and diastolic velocities is prominent even before the development of left ventricular hypertrophy (Charron et al., 1997).

Increased myofilament Ca^2+^ sensitivity, as observed in three *Mybpc3* cardiomyopathy mouse models (*Mybpc3* KO and KI) developed by us and others (Cazorla et al., 2006; Pohlmann et al., 2007; Vignier et al., 2009; Fraysse et al., 2012; Barefield et al., 2014), and in other animal models of HCM (Knollmann et al., 2001; Robinson et al., 2007; Iorga et al., 2008), could be an underlying cause of diastolic dysfunction. This observation has also been made in human HCM samples (Jacques et al., 2008; van Dijk et al., 2009, 2012) and could explain the incomplete relaxation in diastole in *MYBPC3*-associated HCM (and probably other cases associated with an increased Ca^2+^ sensitivity). Additionally, myofilaments with increased sensitivity to Ca^2+^ may act as Ca^2+^ buffers, prolonging the export of Ca^2+^ and relaxation time which could contribute to diastolic dysfunction and arrhythmias (Morimoto et al., 1998; Baudenbacher et al., 2008). These findings support the hypothesis that interventions decreasing myofilament Ca^2+^ sensitivity could reverse the phenotype of HCM and have therapeutic value (Jagatheesan et al., 2007; Alves et al., 2014; Tardiff et al., 2015).

Epigallocatechin-3-gallate (EGCg), a major component of green tea, has been suggested to be effective against cardiovascular diseases. Proposed mechanisms were anti-oxidative, anti-inflammatory, vasorelaxant, and positive inotropic effects (Chyu et al., 2004; Lorenz et al., 2004; Ludwig et al., 2004). Furthermore, it was shown that EGCg lowered myofilament Ca^2+^ sensitivity in a transgenic HCM mouse model expressing a human cardiac troponin T (*TNNT2*, cTnT) mutant (Tadano et al., 2010) and in HCM-associated human cardiac troponin I (*TNNI3*, cTnI) and cTnT mutants in a reconstituted acto-myosin system (Warren et al., 2015; Messer et al., 2016). However, the effects of EGCg were not evaluated in other HCM models associated with mutations in the thick filament of the sarcomere. Since *MYBPC3* is the major disease gene constituting 45% of genetically diagnosed HCM cases (Ho et al., 2015), we used a representative mouse model carrying the human c.772G> A *MYBPC3* mutation (Vignier et al., 2009). This mutation was found in 14% unrelated HCM patients in Tuscany and is associated with a bad prognosis (Richard et al., 2003; Girolami et al., 2006; Ho et al., 2015). These mice exhibit, in addition to left ventricular hypertrophy and decreased fractional area shortening, increased myofilament Ca^2+^ sensitivity, and diastolic dysfunction (Fraysse et al., 2012). We evaluated the acute effects of EGCg on sarcomere shortening and Ca^2+^ transient in intact ventricular myocytes and on force-Ca^2+^ relationship of skinned cardiac muscle strips isolated from KI and wild-type (WT) mice.

## Materials and methods

### Animals

The *Mybpc3* KI cardiomyopathy mouse model was generated by the targeted insertion of a G > A transition on the last nucleotide of exon 6 and maintained on the Black Swiss background (Vignier et al., 2009; Fraysse et al., 2012; Schlossarek et al., 2012, 2014; Gedicke-Hornung et al., 2013; Mearini et al., 2013, 2014; Stöhr et al., 2013; Friedrich et al., 2014; Najafi et al., 2015; Thottakara et al., 2015; Flenner et al., 2016). This study was carried out in accordance with the recommendations of the guide for the care and use of laboratory animals published by the NIH (Publication No. 85–23, revised 2011 published by National Research Council). All experimental procedures were in harmony with the German Law for the Protection of Animals and the protocol was approved by the Ministry of Science and Public Health of the City State of Hamburg, Germany (Org 653).

### Ventricular myocyte preparation

Cardiomyocytes were isolated from WT and KI mouse heart ventricles as previously described (El-Armouche et al., 2007; Pohlmann et al., 2007; Flenner et al., 2016). Mice were anesthetized with CO_2_ and sacrificed by cervical dislocation. Hearts were excised, cannulated via the aorta and installed on a temperature-controlled (37°C) perfusion system. After retrograde perfusion with Ca^2+^-free buffer solution (113 mM NaCl, 4.7 mM KCl, 0.6 mM KH_2_PO_4_, 0.6 mM Na_2_HPO_4_, 1.2 mM MgSO_4_, 12 mM NaHCO_3_, 10 mM KHCO_3_, 30 mM taurine, 5.55 mM glucose, 10 mM 2,3-butanedione monoxime 10 mM HEPES, pH 7.46) for 6.5 min, hearts were digested with 0.075 mg/ml Liberase TM (Roche Diagnostics, Mannheim, Germany) dissolved in buffer solution containing 12.5 μM CaCl_2_ for 7–8 min. Ventricles were disconnected from the atria and minced with forceps to dissociate single cardiomyocytes. Afterwards Ca^2+^ was introduced stepwise up to a concentration of 1 mM.

### Sarcomere shortening and Ca^2+^ transient measurements in intact ventricular myocytes

For contractile analysis only rod-shaped myocytes without membrane blebs, hypercontractile zones, and spontaneous activity showing a stable contraction amplitude and rhythm at 1-Hz pacing frequency (4 ms long 10 V pulses) and 37°C were recorded. Sarcomere shortening and Ca^2+^ transients were recorded using a video-based sarcomere detection system and analyzed with the appendant software (IonWizard; IonOptix; Milton, MA) as described (Flenner et al., 2016). For Ca^2+^ recordings, cells were loaded with 0.6 μM Fura-2-AM and excited at 340 and 380 nm while the emitted light at 510 nm was recorded with a photon multiplier tube. Measurements of contraction and Ca^2+^ transients were first performed by perfusion of the cells in basal buffer (135 mM NaCl, 4.7 mM KCl, 0.6 mM KH_2_PO_4_, 0.6 mM Na_2_HPO_4_, 1.2 mM MgSO_4_, 1.5 mM CaCl_2_, 20 mM glucose, 10 mM HEPES, pH 7.46). When the cells showed stable contraction amplitude, contractile function was recorded. Subsequently, the perfusion was switched to buffer containing different EGCg concentrations (Sigma-Aldrich, 10 nM, 100 nM, 1 μM, 1.8 μM, 3 μM, 10 μM, 30 μM, 100 μM for the concentration-response curve; 1.8 μM for the definite measurements in KI and WT cells) and contractile function was recorded again.

### Skinned ventricular trabeculae force measurements

For the determination of force-Ca^2+^ relationships, trabeculae were prepared from the left ventricular endocardial surface of WT and KI mice as reported before (Flenner et al., 2016). The Ca^2+^-sensitivity of skinned EHT strips was evaluated using a permeabilized fiber test system (1400A; Aurora Scientific). Triton X-100 permeabilized strips of the left ventricle of WT and KI mouse hearts were mounted between a force transducer and a length controller. Trabeculae were stretched above slack length until they developed force in activating solution (pCa 4.5) at 15°C. Subsequently they were exposed to increasing Ca^2+^ concentrations from pCa 9 to pCa 4.5 in EGTA-buffer. Force development was measured in each pCa solution. Measurements were repeated in the presence of 30 μM EGCg after 5 min preincubation in relaxing solution (Flenner et al., 2016). In every second measurement, EGCg was tested first and a control measurement was performed 5 min after EGCg washout to exclude time-dependent loss of force. Data were analyzed using the Hill equation (Hill et al., 1980), with pCa_50_ as the free Ca^2+^ concentration which yields 50% of the maximal force and nH representing the Hill coefficient. The pCa_50_ represents the measure of myofilament Ca^2+^ sensitivity.

### Statistical analysis

Data were expressed as mean±SEM. Comparisons were performed by paired or unpaired Student's *t*-test (effects in intact cardiomyocytes in the absence or presence of EGCg), and with one-way ANOVA, followed by Bonferroni's post-test as indicated in the figure legends (analysis of total, Ser16- and Thr17 phosphorylated phospholamban levels in isolated cells), as indicated in the figure legends. Concentration response curves were fitted to the data points and force-pCa relationship comparison was done by using extra sum-of-squares *F*-test (GraphPad, Prism 6). A value of *P* < 0.05 was considered statistically significant.

## Results

### EGCg (1.8 μM) has no effect on diastolic sarcomere length, but shortens relaxation time in isolated cardiomyocytes

**Figure 1:**
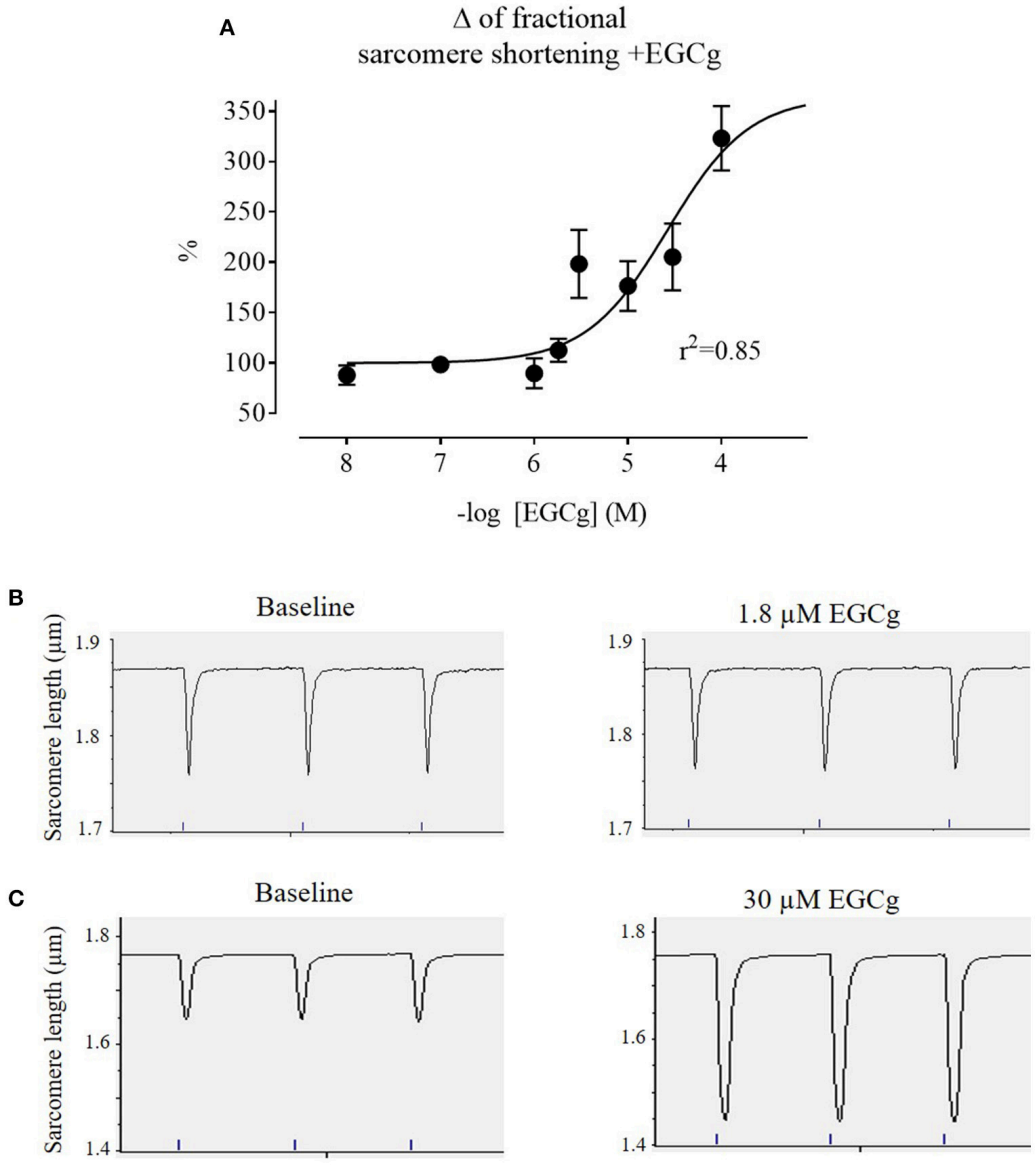
**EGCg effect on ***Mybpc3*** WT cardiac myocyte contractility. (A)** After isolation of ventricular myocytes from adult *Mybpc3* WT mice, paired (before/after EGCg) concentration-response curves were performed. Graph depicts % of fractional sarcomere shortening as readout with increasing EGCg concentrations (10^−8^ = 100 nM, 10^−7^ = 10 nM, 10^−6^ = 1 μM, 10^−5.74^ = 1.8 μM, 10^−5.52^ = 3 μM, 10^−5^ = 10 μM, 10^−4.52^ = 30 μM, 10^−4^ = 100 μM; *n* = 3–9/concentration. Concentrations above ≥3 μM EGCg caused a significant increase in sarcomere shortening. **(B,C)** Representative contractions of a cardiac myocyte at baseline conditions (left) and after 5 min of exposure to 1.8 (**B**, right) or 30 μM EGCg (**C**, right).

EGCg has been reported to concentration-dependently increase contractile function in rodents' cardiac myocytes and hearts (Lorenz et al., 2008; Tadano et al., 2010). HCM patients typically present with a normal or increased ejection fraction, but a diminished diastolic function and incomplete relaxation (Elliott et al., 2014). This is mimicked in cardiac myocytes from *Mybpc3* KI mice, which showed lower diastolic sarcomere length and higher twitch amplitude than WT cardiomyocytes (Fraysse et al., 2012). We aimed at using an EGCG concentration that would not increase contraction amplitude. Therefore, we performed paired concentration-response curves with sarcomere shortening as the readout on isolated cardiac myocytes of *Mybpc3* WT mice with increasing EGCg concentrations ranging from 10^−8^ to 10^−4^ M (Figure 1A). EGCg increased sarcomere shortening in a concentration-dependent manner (curve fit *r*^2^ = 0.85). The positive inotropic effect of EGCg occurred within 5 min of exposure and was reversible by washout (loss of effect after 5 min). The highest concentration of EGCg that did not alter myocyte contractions was 1.8 μM (= 10^−5.74^ M; Figures 1A,B), while EGCg concentrations above ≥3 μM (= 10^−5.52^ M) increased sarcomere shortening (Figures 1A,C).

**Figure 2:**
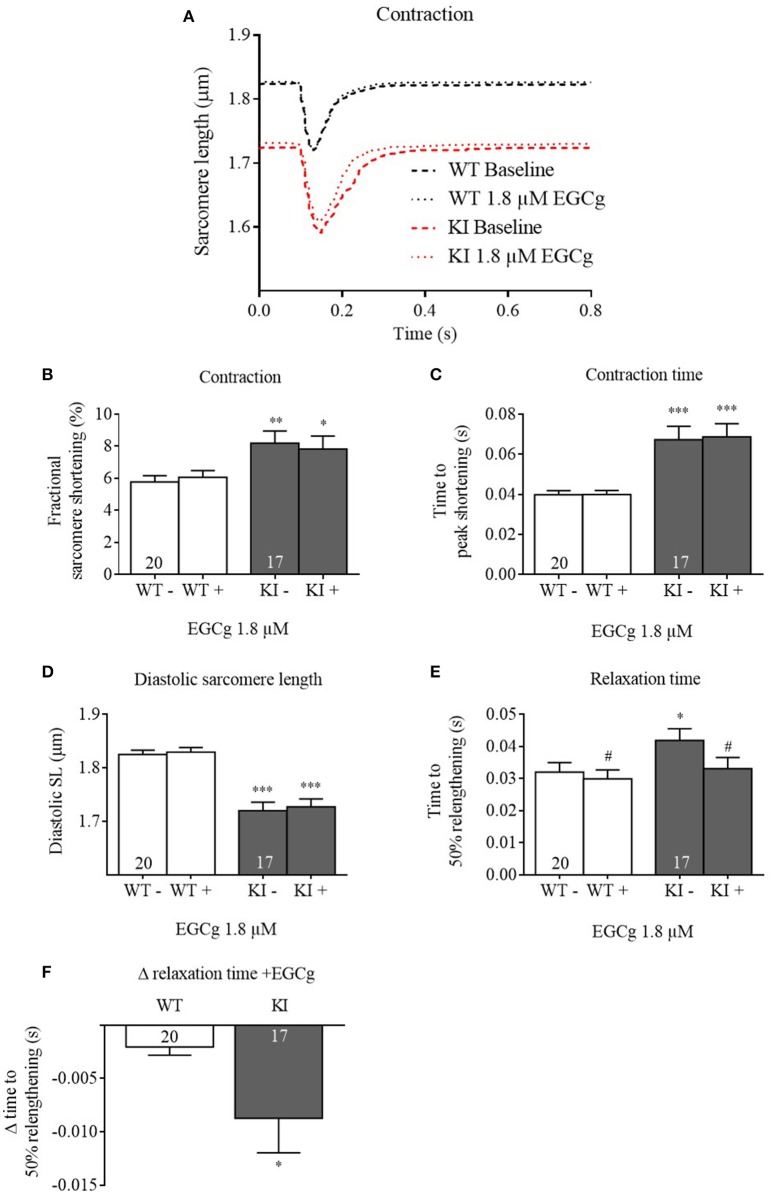
**Contractile parameters of ***Mybpc3*** WT and KI cardiomyocytes before and after treatment with EGCg**. After isolation of ventricular myocytes from adult *Mybpc3* WT and KI mice, paired (before/after EGCg) measurements of contractile function were exerted. **(A)** Averaged sarcomere shortening traces of *Mybpc3* WT (black) and KI (red) cells in baseline and with EGCg. **(B)** Fractional sarcomere shortening, **(C)** contraction time (time from stimulation to peak of contraction), **(D)** diastolic sarcomere length, and **(E)** relaxation time (time from peak of contraction to 50% relaxation) were analyzed. **(F)** Delta of relaxation time (before/after EGCg). ^*^P < 0.05, ^**^P < 0.01 and ^***^P < 0.001 vs. WT in the same condition, unpaired Student's *t*-test; ^#^*P* < 0.05 vs. baseline, paired Student's *t*-test, *n* = 17–22, *N* = 5.

We therefore tested the acute effects of 1.8 μM on isolated cardiac WT and KI myocytes. At baseline and in the absence of Fura-2, KI cardiomyocytes exhibited lower diastolic sarcomere length and longer contraction and relaxation times than WT (Figure 2), recapitulating a relaxation deficit seen in human patients. Application of 1.8 μM EGCg did not affect fractional sarcomere shortening and contraction time (Figures 2B,C). It did not further decrease the pathological diastolic sarcomere length (Figure 2D) but accelerated relaxation, i.e., it lowered relaxation time in both genotypes (Figure 2E). This effect was stronger in KI cells (Figure 2F).

### EGCg (1.8 μM) increases diastolic Ca^2+^ and accelerates Ca^2+^ transient kinetics in isolated cardiomyocytes

**Figure 3:**
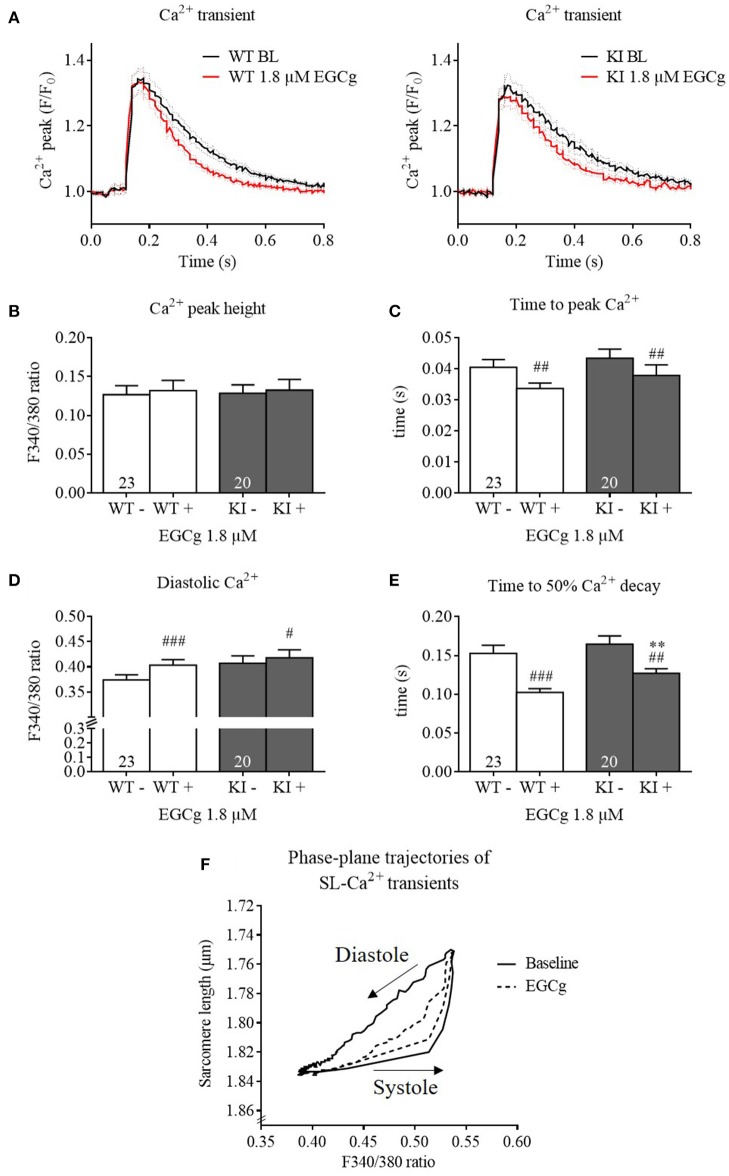
**Ca^**2+**^ transients and kinetics of ***Mybpc3*** WT and KI cardiomyocytes before and after treatment with EGCg**. After isolation of cardiac myocytes from adult *Mybpc3* WT and KI mice, paired (before/after EGCg) measurements of Ca^2+^ transients were performed in Fura-2 loaded cells. **(A)** Averaged Ca^2+^ transients of *Mybpc3* WT and KI cells in baseline and with EGCg. **(B)** Ca^2+^ peak height, **(C)** time to peak Ca^2+^ (from stimulation to peak of 340/380 ratio), **(D)** diastolic Ca^2+^, and **(E)** time to 50% Ca^2+^ decay (from peak of 340/380 ratio to 50% decay) were analyzed. **(F)** Sarcomere length of *Mybpc3* WT cells plotted against the Fura-2 signal ratio F340/380 indicating the Ca^2+^ transient in the absence (black loop) or presence (dotted black loop) of 1.8 μM EGCg, respectively. Loops proceed in a counter-clockwise direction. ^**^P < 0.01 vs. WT in the same condition, unpaired Student's *t*-test; ^#^*P* < 0.05, ^*##*^*P* < 0.01 and ^*###*^*P* < 0.001 vs. baseline, paired Student's *t*-test, *n* = 20–26, *N* = 5. For loops: *n* = 9.

We then investigated whether EGCg influences Ca^2+^ homeostasis and performed Ca^2+^ transient analysis using Fura-2 AM. Contractile parameters of Fura-2-loaded cells were also measured and evaluated, but not represented here, as Fura-2 has a substantial Ca^2+^ buffering effect and therefore interferes with contractile processes. At baseline, KI cardiomyocytes exhibited no difference in Ca^2+^ peak height, diastolic Ca^2+^, time to peak Ca^2+^, and time to 50% Ca^2+^ decay compared to WT cells (Figure 3). Stimulation with EGCg had no influence on Ca^2+^ peak height, but slightly increased diastolic Ca^2+^, and markedly reduced time to peak Ca^2+^ and time to 50% Ca^2+^ decay in both groups. Even though the time to 50% Ca^2+^ decay was longer in KI cells in the presence of EGCg, the delta was not different to WT (WT −0.05 ± 0.008 s vs. KI −0.04 ± 0.0098 s, *P* = 0.33, Student's *t*-test). Plotting the sarcomere length against the F340/380 ratio of Fura-2 in WT cells showed that with 1.8 μM EGCg, the descending phase of the relation between the F340/380 ratio and the sarcomere length was shifted to the right, and the relaxed state of the sarcomere length in diastole was reached at higher F340/380 ratios, resulting in smaller loops (Figure 3F). Opposite results, a left-shift of the loop, had been reported with the Ca^2+^ sensitizer CGP-48506 (Wolska et al., 1996).

### EGCg (30 μM) decreases myofilament Ca^2+^ sensitivity to a greater extent in KI than in WT skinned ventricular trabeculae

**Figure 4:**
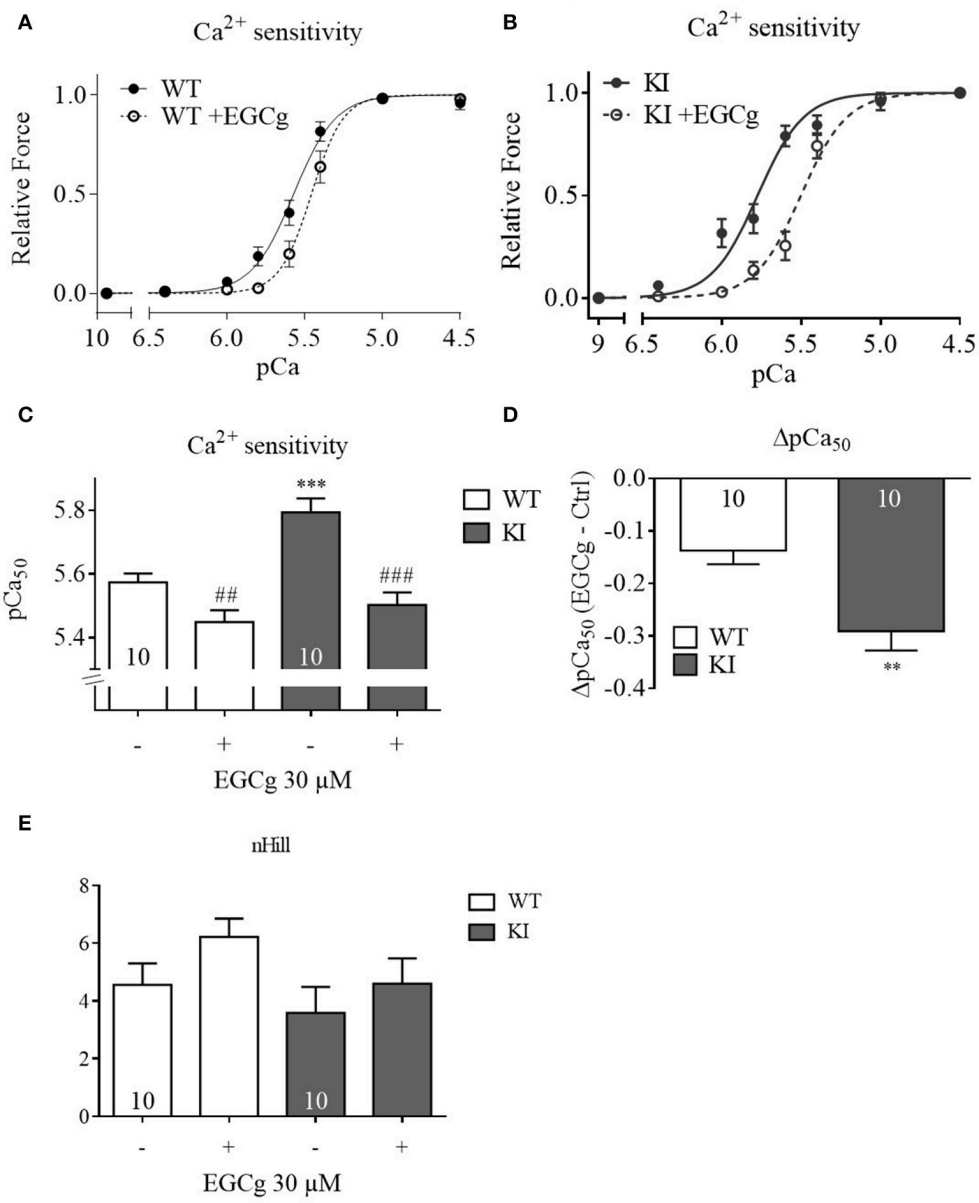
**Force-Ca^**2+**^ relationship of permeabilized cardiac muscle strips of WT and KI mice before and after treatment with 30 μM EGCg**. Force-Ca^2+^ concentration in **(A)** WT strips, **(B)** KI strips. (**C)** The pCa_50_ represents the measure of myofilament Ca^2+^-sensitivity. **(D)** Delta of pCa_50_before and after EGCg. **(E)** nHill coefficient±EGCg. ^**^P < 0.01 and ^***^P < 0.001 vs. WT in the same condition, unpaired Student's *t*-test; ^*##*^*P* < 0.001 and ^*###*^*P* < 0.001 vs. baseline, paired Student's *t*-test, concentration response curves were fitted to the data points and curve comparison was done by using extra sum-of-squares *F*-test; *n* = 10; *N* = 6 mice/genotype.

EGCg has been reported to decrease myofilament Ca^2+^ sensitivity in three HCM models expressing either a *TNNT2* or *TNNI3* mutation (Tadano et al., 2010; Warren et al., 2015; Messer et al., 2016). To assess whether the EGCg effects in intact cells resulted from a decrease in myofilament Ca^2+^ sensitivity, we measured force-pCa relationships in skinned ventricular trabeculae from WT and KI mice. At baseline and as observed before (Fraysse et al., 2012; Flenner et al., 2016), skinned KI trabeculae showed a higher pCa_50_than WT trabeculae, indicating higher myofilament Ca^2+^ sensitivity (Figures 4A–C). We first tested a concentration of 10 μM on *Mybpc3* KI muscle strips but did not observe any effect (data not shown). Since other groups had reported that EGCg concentrations below 30 μM had no effect on Ca^2+^ sensitivity we also used 30 μM (Tadano et al., 2010; Robinson et al., 2016). Incubation with 30 μM EGCg shifted the force-Ca^2+^ relationship to the right resulting in a lower pCa_50_ in both genotypes (Figures 4A–C), which indicates myofilament Ca^2+^ desensitization. As observed in the myocyte experiments, the effect of EGCg was stronger in skinned KI than WT muscle strips (Figure 4D). The nHill coefficient did not differ between the genotypes neither with nor without EGCg (Figure 4E).

## Discussion

One major feature of HCM is a compromised diastolic function with an incomplete relaxation (Elliott et al., 2014). Increased myofilament Ca^2+^ sensitivity as seen in HCM patients and in several mouse models of HCM (Morimoto et al., 1998; Robinson et al., 2007; Huke and Knollmann, 2010; Kimura, 2010; Fraysse et al., 2012; Moore et al., 2012; van Dijk et al., 2012; Barefield et al., 2014; Flenner et al., 2016) could contribute to diastolic dysfunction. Recent findings advocate a potential therapeutic role for EGCg in HCM since it lowered the increased myofilament Ca^2+^ sensitivity in a transgenic HCM mouse model expressing a human cardiac troponin T (*TNNT2*, cTnT) mutant (Tadano et al., 2010) and in HCM-associated human cardiac troponin I (*TNNI3*, cTnI) and cTnT mutants in a reconstituted acto-myosin system (Warren et al., 2015; Messer et al., 2016). The aim of this study was to evaluate whether EGCg has beneficial effects in another thick filament model of HCM, carrying a human mutation in the thick filament protein gene *MYBPC3*. The main findings of this study were: 1. At baseline and in the absence of Fura-2, KI cardiomyocytes exhibited higher fractional sarcomere shortening, lower diastolic sarcomere length, longer contraction and relaxation times than WT cells, without differences in Ca^2+^ transient amplitude and kinetics. 2. EGCg had no effect on sarcomere shortening or diastolic sarcomere length, but it accelerated relaxation and Ca^2+^ transient decay in *Mybpc3* WT and KI cardiomyocytes. 3. EGCg induced myofilament Ca^2+^ desensitization in permeabilized left ventricular trabeculae isolated from *Mybpc3* WT and KI mouse hearts. 4. EGCg effects on relaxation time and myofilament Ca^2+^ sensitivity were more pronounced in KI cells and muscle strips, respectively.

EGCg is a major component of green tea and has been reported to have beneficial effects in a variety of diseases (Peng et al., 2011; Brückner et al., 2012; Ortsäter et al., 2012). Suggested mechanisms in the context of cardiovascular diseases are anti-oxidative, anti-inflammatory, vasorelaxant and positive inotropic effects (Chyu et al., 2004; Lorenz et al., 2004; Ludwig et al., 2004). Since HCM patients typically present with a normal or even increased ejection fraction, but with a diminished diastolic function (Elliott et al., 2014), mimicked in *Mybpc3* KI mice (Fraysse et al., 2012), we intended to apply the highest concentration that would not increase sarcomere shortening (1.8 μM) to evaluate its effects on the impaired relaxation in KI myocytes. Similar to Lorenz et al. and Tadano et al. (Lorenz et al., 2008; Tadano et al., 2010), who reported a positive inotropic effect between 2.5 and 5 μM in mouse hearts and rat myocytes, we observed an increase in fractional sarcomere shortening at concentrations above 3 μM. Although we observed no effect on diastolic sarcomere length in WT cells at 1.8 μM EGCg, we speculated that this concentration could have an effect in KI cells. This was not the case. Nevertheless, 1.8 μM EGCg reduced relaxation time, and this effect was more prominent in KI cells, whereas the effects on diastolic Ca^2+^ and Ca^2+^ kinetics did not differ between the genotypes. The increase in diastolic Ca^2+^ could be explained by an inhibiting effect on the Na^+^/Ca^2+^ exchanger (NCX), as reported with 10 nM (Feng et al., 2012) and 2.5 μM (Lorenz et al., 2008). The faster time to peak Ca^2+^ is probably due to EGCg effects on the ryanodine receptor. Indeed, it has been shown that EGCg activates the ryanodine receptor at 10 nM in sarcoplasmic reticulum (SR) vesicles isolated from rabbit left ventricles (Feng et al., 2012) and in the range of 1 nM–20 μM in junctional SR vesicles isolated from skeletal muscle (Najafi et al., 2015). At a concentration of 1.8 μM, EGCg had no effect on PLB Ser16/Thr17 phosphorylation (Supplemental Figure 1). This supports findings of Lorenz et al., who neither observed an effect on PLB Ser16/Thr17 phosphorylation with EGCg (4 μM) nor an influence on EGCg actions on contractility after β1-adrenoceptor inhibition with 3 μM metoprolol (Lorenz et al., 2008). We thus exclude that the acceleration of relaxation and Ca^2+^ kinetics are mediated via the β1-adrenergic pathway. In contrast to Lorenz et al. we did not observe an increased Ca^2+^ peak height in the presence of 1.8 μM EGCg (Lorenz et al., 2008). We therefore assume that at this concentration EGCg does not increase sarcoplasmic reticulum (SR) Ca^2+^ load. Data from canine SR vesicles and HEK293 cell microsomes show that EGCg concentrations > 4.8 μM directly inhibit SR calcium ATPase (Kargacin et al., 2011). Additionally, Feng et al. reported no effect on SERCA in rabbit cardiac and skeletal SR membranes with 1–2 μM EGCg (Feng et al., 2012; Najafi et al., 2015). We therefore rule out that the acceleration of relaxation kinetics in the presence of 1.8 μM EGCg is due to an increased SERCA activity. A plausible contributing reason could be an EGCg-mediated decrease in myofilament Ca^2+^ sensitivity.

Indeed, it has been reported that EGCg lowered the increased myofilament Ca^2+^ sensitivity and improved the diastolic function of isolated working heart preparations from a transgenic HCM mouse model expressing a human *TNNT2* mutation (Tadano et al., 2010). EGCg also restored the increased Ca^2+^ sensitivity of HCM-associated human cTnI and cTnT mutants in a reconstituted acto-myosin system (Warren et al., 2015; Messer et al., 2016). Similar to these previous data, 30 μM EGCg decreased Ca^2+^ sensitivity in our thick myofilament mouse model that carries a frequent HCM mutation in the most frequently mutated gene (Vignier et al., 2009; Fraysse et al., 2012; Ho et al., 2015). It has been suggested that EGCg binding to the C-terminal region of cardiac troponin C (cTnC) alters the interaction between cTnC and cTnI and therefore the sensitivity of the myofilaments to Ca^2+^ (Liou et al., 2008; Robertson et al., 2009). In both intact myocytes and skinned trabeculae, EGCg had a more profound effect on cells and strips of the KI genotype. This could also be related to the longer baseline relaxation time and elevated myofilament Ca^2+^ sensitivity in KI mice. This is similar to a recent study in which we showed that the myofilament Ca^2+^-desensitizing effect of ranolazine was only present in KI, but not in WT muscle strips (Flenner et al., 2016). Since EGCg is not a pure Ca^2+^ desensitizer (Stangl et al., 2007), side effects such as arrhythmias or blood pressure lowering reported in *in vivo* applications would also be expected in WT mice (Alvarez et al., 2006; Bao et al., 2015).

The study has two limitations. (1) The *Mybpc3* KI model shows many HCM features only at the homozygous state. Additionally, *Mybpc3* KI mice present a reduced ejection fraction. These two points are in contrast to the more common findings in HCM patients who present left ventricular hypertrophy, interstitial fibrosis, and diastolic dysfunction with heterozygous mutation states and normal or even supra-normal ejection fraction. (2) The Ca^2+^-desensitizing effect of EGCg on the permeabilized trabeculae was only detected at 30 μM, which is higher than the concentrations used in the intact myocyte experiments. This discrepancy has also been observed before (Tadano et al., 2010; Robinson et al., 2016). It should be kept in mind that in permeabilized cardiac muscle fibers the effective concentrations of the Ca^2+^-sensitizers pimobendan and EMD57033 were also reported to be much higher than those estimated *in vivo* (Fujino et al., 1988; Solaro et al., 1993; Chu et al., 1999), proposing that drugs could have lower potency in permeabilized cardiac muscle preparations than *in vivo*, since important components enhancing drug uptake or function such as membrane transporters or the SR could be disturbed in their function or missing after muscle strip skinning.

## Conclusion

EGCg accelerated relaxation and Ca^2+^ transient decay in *Mybpc3* WT and KI cardiomyocytes, which seems to be partly due to Ca^2+^ desensitization of the myofilaments. We show for the first time that EGCg is also effective in a thick filament mutation mouse model. In support of other studies (Tadano et al., 2010; Warren et al., 2015; Messer et al., 2016), this confirms that EGCg belongs to a new class of Ca^2+^ antagonists which have a very favorable functional profile acting directly on the Ca^2+^ regulatory system of cTnC. Further efforts should be made to develop a new generation of Ca^2+^ desensitizers based on the green tea catechin EGCg with even more potency and specificity to avoid potential side effects such as arrhythmias or lowering of blood pressure that could also affect healthy individuals.

## Author contributions

FWF: conception and design of research, management of the mouse cohorts, execution of experiments, analysis, and interpretation of data, figure preparation, drafting of the manuscript. FF: isolation and treatment of cardiac myocytes, execution of experiments, interpretation of data, figure preparation, discussion of the manuscript draft. MN: isolation and treatment of cardiac myocytes, execution of experiments. TE: interpretation of data, discussion of the manuscript draft. LC: conception and design of research, analysis, and interpretation of data, drafting of the manuscript. All authors critically discussed the results, and reviewed and approved the manuscript before submission.

## Funding

This work was supported by the DZHK (German Centre for Cardiovascular Research) and the Deutsche Stiftung für Herzforschung (F/28/12).

### Conflict of interest statement

The authors declare that the research was conducted in the absence of any commercial or financial relationships that could be construed as a potential conflict of interest.
