# Supplementary material for: Epigallocatechin-3-Gallate Accelerates Relaxation and Ca2+ Transient Decay and Desensitizes Myofilaments in Healthy and Mybpc3-Targeted Knock-in Cardiomyopathic Mice
Source: Front Physiol. 2016 Dec 5;7:607. doi: 10.3389/fphys.2016.00607 (PMC5136558; doi:10.3389/fphys.2016.00607)
Supplement: Supplementary file 1 [file DataSheet1.docx]

Supplementary Material

**Epigallocatechin-3-gallate accelerates relaxation and Ca^2+^ transient decay and desensitizes myofilaments in healthy and *Mybpc3*-targeted knock-in cardiomyopathic mice**

Felix W. Friedrich^1,2*^, Frederik Flenner^1,2^, Mahtab Nasib^1,2^, Thomas Eschenhagen^1,2^, Lucie Carrier^1,2*^

*** Correspondence:**

Felix W. Friedrich, f.friedrich@uke.de or

Lucie Carrier, l.carrier@uke.de

# Supplementary Data

## Material and Methods: Post-translational modifications of cardiac proteins

Isolated adult ventricular myocytes were kept in modified Tyrode’s solution and stimulated with 1.8 µM EGCg for 5 min at room temperature. After treatment, cells were collected and homogenized in protein lysis buffer (30 mM Tris pH 8.8, 5 mM EDTA, 30 mM NaF, 3% SDS, 10% glycerol) and proteins were separated via SDS-PAGE using a 12% acrylamide/bisacrylamide (29:1) gel. Separated proteins were transferred to a polyvinylidene fluoride membrane at 300 mA for 90 min and then incubated with different antibodies which were detected by chemiluminescence as described previously ([Friedrich, Sotoud et al. 2015](#_ENREF_14)). The following antibodies and dilutions were used: S6 ribosomal protein (S6, rabbit, monoclonal, Cell Signaling) 1:1,000, phospholamban (PLB, mouse, monoclonal, Badrilla) 1:2,000, Ser-16-PLB (rabbit, polyclonal, Badrilla) 1:5,000, Thr-17-PLB (rabbit, polyclonal, Badrilla) 1:5,000.

## Results:

Analysis of total and Ser16/Thr17 phosphorylated (pSer16/pThr17) PLB levels in adult ventricular cardiomyocytes showed no difference in total or pSer16/pThr17 PLB levels in untreated WT and KI cells and no difference in total or pSer16/pThr17 PLB between untreated and EGCg-treated cells in both genotypes excluding an increased PLB phosphorylation as the mechanism behind the EGCg effects.

# Supplementary Figure


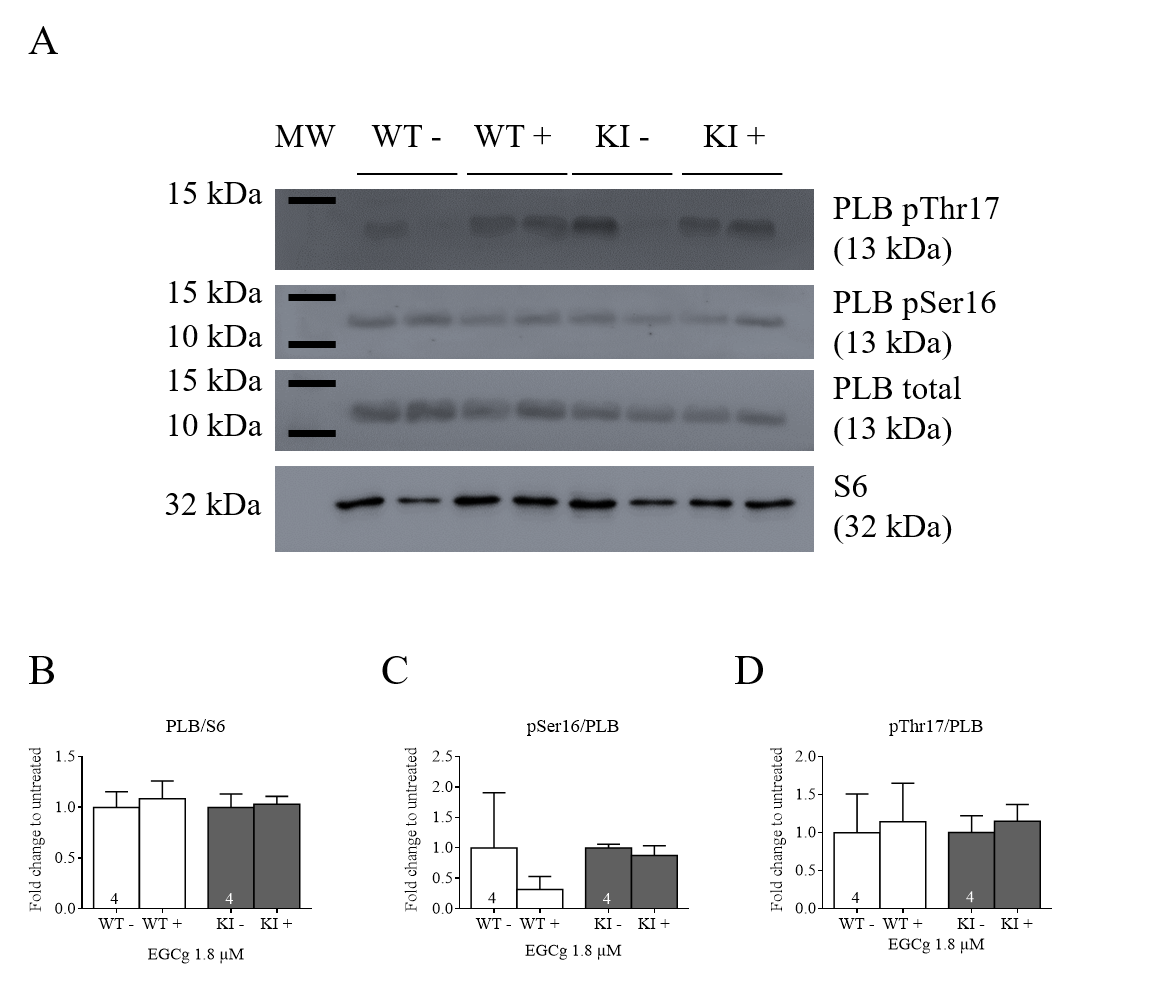


**Supplementary Figure 1.** **Total and Ser16-(pSer16) and Thr17-(pThr17) phosphorylated phospholamban levels in cardiomyocytes of WT and KI mice ±1.8 µM EGCg.** Isolated ventricular myocytes from adult *Mybpc3* WT and KI mice were stimulated ±1.8 µM EGCg for 5 min at room temperature. After cell harvest, proteins were isolated and a Western Blot was performed. (A) Representative Western Blot. PLB total and pSer16Tp/Thr17 values were normalized to S6 ribosomal protein (S6) as loading control. (B) Total PLB values were related to S6 values, (C) pSer16/S6 and (D) pThr17/S6 values were related to total PLB/S6 values. WT levels are illustrated in white, KI levels in gray, normalized to untreated samples, n=4.
